# Case Report: Ischaemic appendicitis post mesenteric biopsy

**DOI:** 10.12688/f1000research.7209.1

**Published:** 2016-01-04

**Authors:** Marianna Zukiwskyj, June Tun, Shashank Desai

**Affiliations:** 1 Department of General Surgery, Ipswich Hospital, Ipswich, IP4 5PD, UK

**Keywords:** Ischaemic appendicitis, biopsy, Carcinoma, mesenteric lymph node

## Abstract

A common indication for laparoscopic mesenteric lymph node biopsy is to provide a tissue diagnosis in the absence of palpable peripheral nodes via a minimally invasive approach.  There are no reports to date of ischaemia to the appendix as a complication of this procedure.   We report the case of a 34-year-old lady who underwent a mesenteric biopsy for a lesion found incidentally on CT to investigate longstanding abdominal pain, and 2 days later required an appendicectomy for ischaemic appendicitis.

## Introduction

Appendicitis is the most common intra-abdominal surgical emergency. The classical pathophysiology of appendicitis is that of obstruction of the lumen and subsequent bacterial overgrowth ^3, 8^. Appendicitis arising as a complication of a surgical procedure has not been reported in the literature.

A common indication for mesenteric lymph node biopsy, in the setting of enlarged mesenteric nodes or a mesenteric mass on imaging, is to provide a tissue diagnosis in the absence of palpable peripheral nodes via a minimally invasive approach ^1, 5^. Where this cannot by achieved by percutaneous techniques, laparoscopic mesenteric biopsy has been shown to be a safe and effective alternative, when compared with open techniques ^1, 2, 5^. As laparoscopy is a minimally invasive technique, the reduction in wound healing time decreases the delay to therapeutic interventions such as chemotherapy ^2^. Reasons for conversion to laparotomy include adhesions, poor intra-operative exposure and bleeding ^1^. There are no reports to date of ischaemia to the appendix as a complication of this procedure.

We describe a case of appendicitis in the post operative period in a 34-year-old lady who underwent a mesenteric biopsy of a lesion found incidentally on CT.

## Case report

A 34-year-old Caucasian lady, with no significant medical history, presented to her local GP with persistent epigastric pain and nausea. Investigation of the pain included an abdominal CT, which revealed an incidental mass in the right iliac fossa measuring 17mm suspicious for a carcinoid tumour, and no other features of concern or metastatic disease. She underwent a diagnostic laparoscopy and excisional biopsy of this mesenteric mass near the junction of the ileum and caecum, with a Harmonic scalpel. She had an uneventful recovery, and was discharged day 1 postoperatively. She later presented to the emergency department on the same day of discharge, with worsening abdominal pain and fevers. She was taken back to theatre the following day and was found to have a necrotic appendiceal tip, and underwent an appendicectomy.

The histology from the mesenteric biopsy showed a 10×15mm nodule of carcinoid tumour, as well as 2 of 4 lymph nodes containing metastases. The histology from the appendicectomy showed extensive mucosal necrosis, but no evidence of malignancy.

The patient has since undergone an open, uncomplicated right hemicolectomy which showed an 8mm grade 1 neuroendocrine tumour at the region of the ileocaecal valve with clear margins and 11 lymph nodes negative for metastatic disease. She is currently under the care of an oncologist and receiving lanreotide 60–120mg monthly, the duration of which is currently ongoing.

## Discussion

Appendicitis due to primary ischaemia, or as a complication of mesenteric biopsy, is not described in the literature. Acute torsion of the appendix and subsequent appendicitis has been described infrequently in the paediatric population ^4^. The appendiceal artery is the terminal branch of the ileocolic artery, and runs in the free edge of the mesoappendix. Varying descriptions of anatomical locations and incidence of accessory appendiceal arteries are described in the literature ^6^.

We hypothesise that this presentation of acute appendicitis on the second post-operative day following mesenteric biopsy was secondary to inadvertent vascular injury, and subsequent ischaemia, given the proximity of the initial operative field to the ileocaecal junction. One possible mechanism is that appendiceal artery, or a possible aberrant course of the appendiceal artery or accessory appendiceal artery, was not identified during initial laparoscopic survey and was divided during dissection. Another possible mechanism is that the appendiceal artery was indirectly affected by secondary thermal injury during harmonic dissection of the mesenteric lesion. Whilst uncommon, unexpected thermal injuries to surrounding structures have been reported during use of Harmonic dissection ^7^.

## Conclusion

Laparoscopic mesenteric biopsies are becoming increasingly more common as histological diagnosis is required to confirm disease processes. This case illustrates an unusual complication of a procedure often viewed as straightforward and highlights the diligence required during mesenteric dissection to prevent inadvertent injury to nearby structures, as well as the possibility of unusual circumstances resulting in unexpected injuries when using the Harmonic scalpel as an energy source for dissection.

## Consent

Consent for publication of information from this clinical case was sought and obtained from the patient.
