# Supplementary material for: Case Report: Ischaemic appendicitis post mesenteric biopsy
Source: F1000Res. 2016 Jan 4;5:3. [Version 1] doi: 10.12688/f1000research.7209.1 (PMC4752020; doi:10.12688/f1000research.7209.1)
Supplement: Peer review history

# Peer review: Referee response for version 1

**DOI:** 10.5256/f1000research.7765.r11815

**Published:** 2016-02-11

**Authors:** Brian Keith Bednarski

**Affiliations:** 1 Department of Surgical Oncology, Division of Surgery, The University of Texas MD Anderson Cancer Center, Houston, TX, USA

The authors present a case report of a complication from a laparoscopic mesenteric excisional biopsy. Following an excision of an suspected carcinoid node in the mesentery, the patient suffered an ischemic event to the appendix.

My comments/suggestions for the article are as follows:

1. The authors describe that the indications for the laparoscopic excision are for those areas not amenable to percutaneous biopsy. This should also include lesions that have failed image guided biopsy. To that end, please comment on the patient's course in that regard. Did they have an attempted biopsy? Did they have a biopsy that failed to yield the diagnosis?
2. Did the patient have a colonoscopy? With a suspicious carcinoid, an endoscopic evaluation should be considered to assess for primary tumor.
3. Also, please comment on the intraoperative findings in more detail. Carcinoid tumors, and specifically its associated lymphadenopathy, have a very distinctive appearance that can clue in the operative surgeon to the diagnosis. Did you consider intraoperative pathologic assessment? With the possibility of completing the hemicolectomy in the first operation?
4. Lastly, I agree with the other review that additional information regarding recommendations/suggestions to avoid mesenteric vascular injury during these procedures would be helpful.

I have read this submission. I believe that I have an appropriate level of expertise to confirm that it is of an acceptable scientific standard.

# Peer review: Referee response for version 1

**DOI:** 10.5256/f1000research.7765.r12127

**Published:** 2016-02-01

**Authors:** Heidi K Chua

**Affiliations:** 1 Department of Surgery, Mayo Clinic, Rochester, MN, USA

Drs. Zukiwsky, Tun and Desai has described ischemia as a potential complication of mesenteric biopsy.  This certainly highlights the need to be cautious in performing what might be considered a minor procedure. I do have 2 requests:

1. Can they be more descriptive in how the actual excisional biopsy was done - was there an attempt to identify the vascular supply to the nearby structures?
2. Can they suggest ways of preventing ischemic injuries while performing similar procedure?

I have read this submission. I believe that I have an appropriate level of expertise to confirm that it is of an acceptable scientific standard.
